# Application of the Remote Interaction Effect and Molecular Imprinting in Sorption of Target Ions of Rare Earth Metals

**DOI:** 10.3390/polym14020321

**Published:** 2022-01-13

**Authors:** Talkybek Jumadilov, Ruslan Kondaurov, Aldan Imangazy

**Affiliations:** 1 Laboratory of Synthesis and Physicochemistry of Polymers, JSC “Institute of Chemical Sciences after A.B. Bekturov”, Sh. Valikhanov St. 106, Almaty 050010, Kazakhstan; jumadilov_kz@mail.ru (T.J.); imangazy.aldan@mail.ru (A.I.); 2 Department of Chemistry and Technology of Organic Substances, Natural Compounds and Polymers, Al-Farabi Kazakh National University, Al-Farabi Ave. 71, Almaty 050040, Kazakhstan

**Keywords:** rare earth metals, sorption, separation, remote interaction of macromolecules, molecular imprinting

## Abstract

The goal of the present work is a comparative study of the effectiveness of the application of intergel systems and molecularly imprinted polymers for the selective sorption and separation of neodymium and scandium ions. The following physico-chemical methods of analysis were used in this study: colorimetry and atomic-emission spectroscopy. The functional polymers of polyacrylic acid (hPAA) and poly-4-vinylpyridine (hP4VP) in the intergel system undergo significant changes in the initial sorption properties. The remote interaction of the polymers in the intergel system hPAA–hP4VP provides mutual activation of these macromolecules, with subsequent transfer into a highly ionized state. The maximum sorption of neodymium and scandium ions is observed at molar ratios of 83%hPAA:17%hP4VP and 50%hPAA:50%hP4VP. Molecularly imprinted polymers MIP(Nd) and MIP(Sc) show good results in the sorption of Nd and Sc ions. Based on both these types of these macromolecular structures, principally new sorption methods have been developed. The method based on the application of the intergel system is cheaper and easier in application, but there is some accompanying sorption (about 10%) of another metal from the model solution during selective sorption and separation. Another method, based on the application of molecularly imprinted polymers, is more expensive and the sorption properties are higher, with the simultaneous sorption of the accompanying metal from the model solution.

## 1. Introduction

Nowadays, one of the elements highly in demand for industrial use is rare earth metals (REMs). REMs can be named as a critical component in almost all important technologies, which, in turn, drive the modern industrial development across the world. REMs are represented by 15 elements of the lanthanoid group (from La to Lu) plus two elements: Sc and Y; these metals can be considered as a unique row of elements that are used in many areas: lanthanum (battery alloys, metal alloys, auto catalysts, petroleum refining, polishing powders, glass additives, phosphors, ceramics, and optics); cerium (battery alloys, metal alloys, auto catalysts (emissions control), petroleum refining, polishing powders, glass additives, phosphors, and ceramics); praseodymium (battery alloys, metal alloys, auto catalysts, polishing powders, glass additives, and coloring ceramics); neodymium (permanent magnets, battery alloys, metal alloys, auto catalysts, glass additives, and ceramics); promethium (watches, pacemakers, and research; promethium is a radioactive metal, which has no stable isotopes; it is present in the crust of Earth in low quantities); samarium (magnets, ceramics, and medical radiation treatment (cancer diseases)); europium (phosphors); gadolinium (ceramics, nuclear energy, and medicine (magnetic resonance imaging and X-rays)); terbium (fluorescent lamp phosphors and magnets (especially for high temperatures and defense); dysprosium (permanent magnets); holmium (permanent magnets, nuclear energy, and microwave equipment); erbium (nuclear energy, fiber optic communications, and glass coloring); thulium (X-rays (medical) and lasers); ytterbium (cancer treatment and stainless steel); lutetium (age determination and petroleum refining); yttrium (battery alloys, phosphors, and ceramics); and scandium (high-strength, low-weight aluminum–scandium alloys). The increased importance of REMs over the last 100 years is due to their unique properties, which have no existing analogues. This fact can be seen from the rate of annual growth of 13.7% (expected growth between 2017 and 2021) for the global REM market [1,2,3,4,5].

REMs not only replace each other in the structure of the minerals but also occur within different minerals’ structures in the same deposit [6]. As mentioned above, most of the REMs are lanthanoids and their chemical properties are similar. These metals occur together within minerals in varying quantities [7]. The similarity of chemical properties of the REMs creates serious difficulties in their separation after extraction from the minerals where they are found [8,9]. The process of separation of one targeted REM from the amount is difficult, environmentally challenging, and expensive, wherein over 1% of the REM is recycled owing to the many challenges of collecting various end products and separating the REM from other metals/contaminants [10,11]. The main focus for investments in recycling is applications of REMs, such as in magnets, in which the economies of scale allow it. The REM market is relatively small and can be easily disrupted. The main factors that can impact the market are the increase of REM production from existing mines, development of mine prospects advanced during price spikes, research and development efforts focused on improving REM recoveries, recycling, substitution, alternate sources of REMs, and governmental policies [12,13,14].

Hydrometallurgical solutions in various branches of industry have complex chemical composition, which is the limiting stage for the efficient sorption of the target REM ions by ion-exchange resins [15,16,17,18]. Existing ion exchangers have selectivity to a certain metal ion, the first problem showing that the sorption of each ion of REM requires a certain ion-exchange resin. Another drawback of this type of macromolecular structure is its regeneration process, which assumes continuous washing of the ion exchangers, first with acids and then with distilled water for renewing the exchange capacity to initial values after each cycle of sorption of the target metal ions [19,20,21,22,23].

One of the proposed analogues to existing sorption technologies is using the remote interaction effect for the selective sorption of targeted REM ions [24,25]. Functional polymers in intergel systems undergo mutual activation, with further transfer into a highly ionized state, resulting in a significant increase in the initial sorption properties. The use of remote interaction of functional macromolecules has some advantages over the existing sorption methods:

(1) Each intergel system can be efficiently used for the selective sorption of several REM ions by varying polymer molar ratios in it.
(2) The mutually activated macromolecules can be used as independent sorbents.
(3) The high ionization degree of the components in intergel systems leads to significant growth in sorption properties of the initial macromolecules.

Another possible variant of selective sorption of the targeted REM ions can be implemented by using the molecular imprinting technique [26,27,28,29,30,31]. A molecular-imprinted polymer (MIP) is a polymer treated by using a special molecular imprinting technique, which results in the appearance of cavities in the polymer matrix with an affinity for the selected molecular “template” [32,33]. This process typically involves initiating the polymerization of the monomers in the presence of a template molecule, which is subsequently removed, thus leaving complementary cavities. Molecular imprinting is, in fact, an artificial tiny “lock” for a particular molecule, which serves as a miniature “key”. Molecular imprinting is a fairly effective technique for incorporating specific pattern recognition of the analyzed object into polymers [34,35,36,37]. Molecular recognition characteristics of these polymers directly depend on the complementary size and shape of the binding objects, imparted to the polymers by template molecules [38,39,40]. The concept of complementarity includes the correspondence of an imprint to a template both in size and shape and in the presence of complementary functional groups in the imprint that are capable of interacting with the functional groups of the template molecule [41,42,43,44].

In this regard, the goal of the present work is a comparative study of selective sorption of targeted REM ions (on neodymium and scandium) by intergel systems and molecularly imprinted polymers.

## 2. Materials and Methods

### 2.1. Materials

Monomers: acrylic acid (AA), methacrylic acid (MAA), and 4-vinylpyridine (4VP) were purchased from “LaborPharma” Ltd. (Almaty, Kazakhstan); linear polymer: poly-4-vinylpyridine (P4VP); cross-linking agents: N,N′-methylenebis(acrylamide) (MBAA), epichlorohydrin (ECH), and ethyleneglycol dimethacrylate (EGDMA); initiator: azobisisobutyronitrile (AIBN); redox system: K_2_S_2_O_8_–Na_2_S_2_O_3_; solvent: dimethylformamide (DMFA); porogen: toluene; stabilizer: hydroxyethylcellulose (HEC). the rest of mentioned chemicals were purchased from Sigma-Aldrich company (Saint-Louis, MO, USA). Deionized water was used in all experiments (χ = 11 µS/cm; pH = 6.97) was obtained in the DV-1 deionizer (Technokom, Ekaterinburg, Russia).

The monomers of AA and MAA initially undergo vacuum distillation at the following conditions: temperature 75 °C (for AA) and 105 °C (for MAA); rate 2 drops per 4–5 s. The necessity of vacuum distillation is to purify the AA monomers from the inhibitor monomethyl ether of hydroquinone (MeHQ).

### 2.2. Methods

#### 2.2.1. Synthesis of Polymer Hydrogels

##### Synthesis of Polyacrylic Acid Hydrogels

Polymerization of rare-cross-linked AA hydrogels was conducted in the following order: AA monomers were polymerized in the MBAA cross-linking agent in the presence of the redox system K_2_S_2_O_8_–Na_2_S_2_O_3_ in a reactor. The polymerization reaction occurred in the following order: 12 mL of the monomer was put into the volumetric flask (100 mL); after that, 2 drops of the cross-linking agent dissolved in water was added; after that, 1 mL of the initiator was added. After that, the polymerizate was poured into special ampoules (special cylinders without bottom and top) and placed in an oven for 5 h at a temperature of 45–50 °C. During the drying procedure, the ampoules were periodically taken and weighted to the constant weight on analytical scales (importantly, the ampoules were closed when being taken out of the oven to exclude the intake of moisture from the air). The swelling degree of the synthetized hydrogels of PAA was 30.33 g/g.

##### Synthesis of Poly-4-Vinylpyridine Hydrogels

Hydrogels of P4VP were obtained in the following order: initially, linear polymers of P4VP were dissolved in the medium of DMFA and cross-linked by an ECH cross-linking agent under permanent stirring. The polymerization reaction occurred in the following order: a weighed portion of a linear polymer (5 g) was filled with 20 mL of DMFA until the polymer sample was completely dissolved in the solvent; moreover, before the dissolution of the linear polymer, there was a stage of swelling of the polymer in the solvent. After that, 2.5 mL of ECH was added dropwise to the solution, with constant stirring, at a temperature of 60 °C. The swelling degree of the synthetized hydrogels of P4VP was 3.72 g/g.

After the synthesis procedure, the obtained rare-cross-linked hydrogels of PAA and P4VP were purified from unreacted products and soluble polymer fractions by long-term washing (for 14 days) under stationary conditions. The synthesized hydrogels were washed in wash columns. The water was changed 2–3 times a day. The control of the degree of purification was carried out by determining the specific electrical conductivity and pH of water after gel purification. After 14 days, the values of specific electrical conductivity and pH of the wash water remained constant. This indicated that the process of purification of the polymer hydrogels from unreacted products was over. Then the obtained samples of hydrogels were subjected to dispersion by grinding in an analytical mill. Subsequently, the samples of the hydrogels were filtered through a sieve by fractions. For subsequent experiments, samples of PAA and P4VP hydrogels were selected the granule sizes of which were higher than 120 µm and lower than 180 µm.

#### 2.2.2. Preparation of the Intergel System

Previous studies have shown that the intergel system should at least contain two components [45,46]. In the present study, the acidic and basic components of the system were PAA and P4VP hydrogels, respectively. The obtained dispersions of PAA and P4VP hydrogels were put in special polypropylene cells with pores, the cells separated from each other by no more than 100 µm (one cell contained a dispersion of PAA and another a dispersion of P4VP). These cells were impermeable for the macromolecular dispersion but permeable for low-molecular ions. Subsequently, the cells were put in the common solution in a glass; the distance between them was 2 cm. The total amount of the dispersion was 100 mol.% either for the presence of individual polymers or for the presence of intergel pairs. Molar ratios of the polymer hydrogels in intergel systems were taken for convenience. The concentration of the PAA hydrogel decreased from 1.67 mmol/L to 0.28 mmol/L with an increase in the P4VP hydrogel share in the intergel system (molar ratios hPAA:hP4VP 100%:0%–17%:83%), while the concentration of hP4VP increased from 0.28 mmol/L to 1.67 mmol/L with a decrease in the hPAA share.

#### 2.2.3. Synthesis of Molecularly Imprinted Polymers

Molecularly imprinted polymers (MIPs) were synthesized by the suspension polymerization technique. Neodymium and scandium nitrates were chosen as templates. MAA and 4VP were chosen as functional monomers, EGDMA was used as a cross-linking agent, AIBN was used as an initiator, HEC was chosen as stabilizer, and toluene was chosen as a porogen. Polymerization of the MIPs was carried out in deionized water. The composition of the reaction mixture was as follows: template ion:MAA:4VP:EGDMA = 1:2:2:8. The stirring speed was 250 rpm. The reaction was carried out for 15 min at room temperature, then for 6 h at 70 °C in a stream of nitrogen. After polymerization, the resulting MIP particles were thoroughly washed with deionized water and acetone to remove impurities and residues of unreacted monomers. The resulting granules were vacuum-dried for 24 h. To control the selectivity of the MIPs, control samples of cMIPs were synthesized, differing in that no metal template was added during their synthesis. To remove the template from the MIPs, 1 M nitric acid was used, with stirring for 1 h. To completely remove the metals, the washing cycle was repeated 30 times, after which the MIPs were washed with deionized water and dried in vacuum for 24 h.

#### 2.2.4. Sorption Experiments

For the present study, model salt solutions were made: neodymium sulfate hydrate and scandium sulfate hydrate (the concentration was 100 mg/L for each REM salt solution). The macromolecular dispersion (individual polymer hydrogel, intergel system, or molecularly imprinted polymer) was put into the salt solution for 48 h; at specific time intervals (0.5, 1, 2, 6, 24, and 48 h after the beginning of sorption), the aliquots were taken for further analysis of the residual concentration of neodymium/scandium ions.

#### 2.2.5. Laboratory Experiments on Selective Sorption and Separation of Nd and Sc Ions

**Figure 1:**
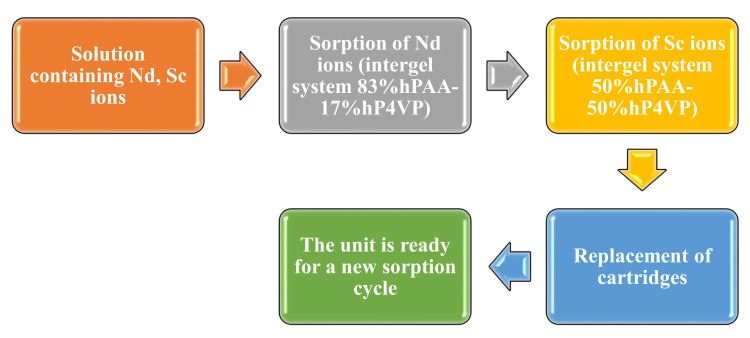
Scheme of Nd and Sc ion sorption and separation using the intergel system hPAA–hP4VP.

Figure 1 shows the scheme of the selective sorption and separation of Nd and Sc ions using the developed unit and the hPAA–hP4VP intergel system. As seen, the scheme involves the following stages:

(1) A solution containing Nd and Sc ions is pumped into the first laboratory unit. The unit is filled with the intergel system 83%hPAA:17%hP4VP for the selective sorption of neodymium ions. One cartridge of the unit contains a dispersion of PAA hydrogel and another a dispersion of P4VP hydrogel. Here, the solution is stored for 48 h. Aliquots are taken at a specific time for further measurement of the residual concentration of the metals’ ions. This stage can be called “sorption of neodymium.”
(2) At this stage, the solution is pumped into the second laboratory unit, which contains a 50%hPAA:50%hP4VP intergel system for the selective sorption of scandium ions for 48 h. The placement of the cartridges inside the unit is similar to that in the first unit (neodymium sorption); one cartridge contains the dispersion of PAA hydrogel and another a P4VP dispersion. Aliquots are taken at the same time for control of the REMs’ residual concentration.
(3) After the sorption of Nd and Sc is fully complete, the cartridges are removed from the units. The new cartridges (four cartridges, two of which contain the intergel system 83%hPAA:17%hP4VP and two of which contain the intergel system 50%hPAA:50%hP4VP) are put into the units, and all is ready for a new cycle of the selective sorption of neodymium and scandium ions.

**Figure 2:**
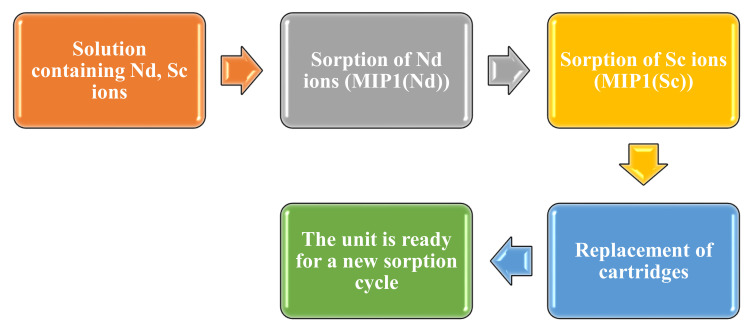
Scheme of Nd and Sc ion sorption and separation using the molecularly imprinted polymers MIP(Nd) and MIP(Sc).

Figure 2 presents a scheme for the sequential selective extraction and separation of Nd and Sc ions using the developed laboratory unit and imprinted structures MIP1(Nd) and MIP1(Sc) as sorbents. This scheme involves two identical developed units placed one after another. The fill of the cartridges in this scheme is quite similar to the case when sorption in the unit is based on intergel systems (Figure 1), but there are some differences: each unit contains only one macromolecular structure: the first unit contains MIP(Nd), and the second unit contains MIP(Sc). From the figure, it can be seen that there are three main stages in the scheme:

(1) The solution containing Nd and Sc ions is pumped into the first unit; one cartridge of the unit is filled with imprinted structure MIP(Nd). In this unit, the solution is stored for 48 h. Aliquots are taken at a specific time for further measurement of the residual concentration of the metals’ ions. The sorption of neodymium occurs at this stage.
(2) Further occurrence of the sorption and separation process proposes that the model solution is pumped to the second unit, one cartridge of which contains imprinted structure MIP(Sc). Aliquots are taken at the same time for control of the REMs’ residual concentration.
(3) After the sorption process of Nd and Sc, the cartridges with MIP(Nd) and MIP(Sc) are removed from the units. Two new cartridges with MIP(Nd) and MIP(Sc) are put into the units, and all is ready for a new cycle of the selective sorption of Nd and Sc ions.

#### 2.2.6. Determination of Initial Electrochemical Properties

For determination of the electric conductivity, the conductometer Expert 002 (Econics-expert, Moscow, Russian, Federation) was used. Measurement of pH values was performed on pH-meter 780 Metrohm (Metrohm, Herizau, Switzerland). These electrochemical properties were controlled during purification of the synthetized polymers (polymer hydrogels, MIPs). Analytical scales Shimadzu AY120 (Shimadzu, Kyoto, Japan) were used for the measurement of the weight of the synthetized polymer hydrogels and further determination of the swelling degree.

#### 2.2.7. Determination of the Residual Concentration of the REM’s Ions

The photocolorimeter KFK-3KM (Unico Sys, Saint-Petersburg, Russian, Federation) was used for determination of the optical density of the REM’s salt solutions. It should be noted that the concentration was determined on the ICP-OES spectrometer 8300 ICP-OES (Perkin Elmer, Waltham, MA, USA).

### 2.3. Calculation of Parameters

The swelling degree of the synthetized rare-cross-linked polymer hydrogels of PAA and P4VP was calculated in accordance with the equation

(1) $$\alpha \frac{m_{2}-m_{1}}{m_{1}}$$

where m_1_ is the mass (g) of the dry polymer hydrogel and m_2_ is the mass (g) of the swollen polymer hydrogel.

Based on the residual concentration after the sorption of Nd and Sc ions, the following sorption parameters were calculated:

(1) Sorption degrees of Nd^3+^ or Sc^3+^ ions:

(2) $$\eta =\frac{{\text{C}}_{0}-{\text{C}}_{e}}{{\text{C}}_{0}}\times 100\%$$

where C_0_ is the initial concentration (mg/L) of the REM’s ions and C_e_ is the initial equilibrium concentration (mg/L) of the REM’s ions.

(2) Dynamic exchange capacity of the polymer structures:

(3) $$Q=\frac{m_{\mathrm{sorbed}}}{m_{\mathrm{sorbent}}}$$

where m_sorbed_ is the mass (mg) of the sorbed REM’s ions and m is the macromolecule’s portion (g). If there are two macromolecules in the corresponding salt solution (presence of the intergel system), this value is determined as the sum of total weight of each macromolecule.

(3) Growth in the sorption parameters (sorption degree/dynamic exchange capacity):

(4) $$\omega_{i}=\frac{P_{i}}{P_{0}}\times 100\%-100\%$$

where P_i_ is the sorption parameter (sorption degree or dynamic exchange capacity) of the intergel system or MIPs at a specific time and P_0_ is the sorption parameter (sorption degree or dynamic exchange capacity) of the PAA or P4VP hydrogel at the same time.

(4) Mean growth in the sorption parameters:

(5) $$\varpi =\frac{\omega_{\eta }+\omega_{Q}}{2}$$

where ω_η_ is the growth in the sorption degree (%) at a specific time and ω_Q_ is the growth in the sorption capacity (%) at the same time.

## 3. Results and Discussion

**Figure 3:**
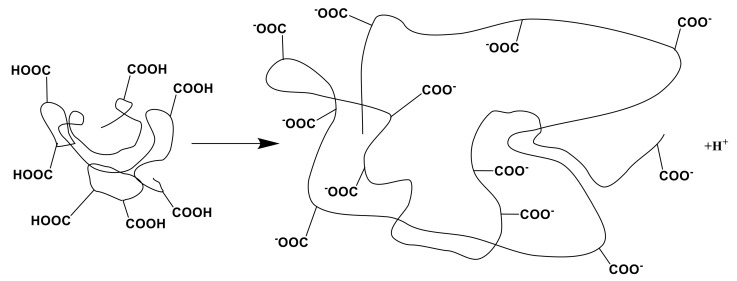
Dissociation of the polyacrylic acid hydrogel.

The remote interaction effect leads to significant changes in the initial properties of the macromolecules in the intergel systems due to changes in the structures of the polymers, wherein direct contact between interacting polymers is absent. Remote interaction of rare-cross-linked polymer hydrogels is accompanied by the following reactions:

(1) Dissociation of -COOH groups of the PAA hydrogel (Figure 3):

Initially, ionization occurs along with formation of ionic pairs; subsequently, the ionic pairs partially dissociate on separate charged particles.

(2) Ionization and partial dissociation of the heteroatom of the pyridine ring (nitrogen atom):

≡N + H_2_O → ≡NH^+^ …OH^–^ → ≡NH^+^ + OH^–^

**Figure 4:**
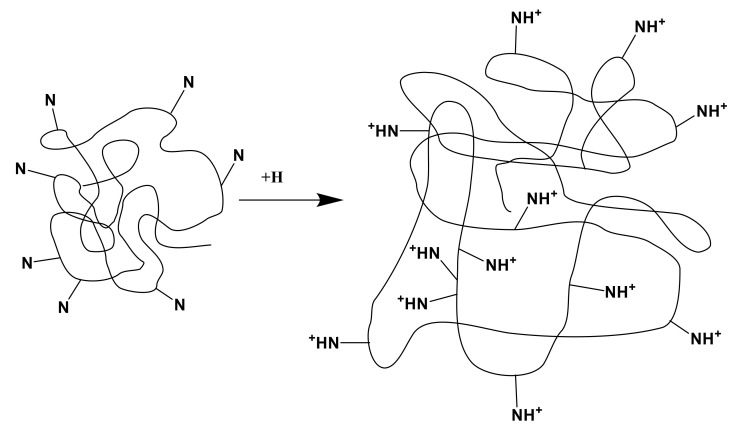
Association of protons by the heteroatoms of the P4VP hydrogel.

(3) Further interaction provides binding of protons cleaved from carboxyl groups by the heteroatoms of the pyridine ring (Figure 4):

(4) Formation of water molecules due to the interaction of H^+^ and OH^–^ ions (right for equimolar concentrations of protons and hydroxyl ions):

H^+^ + OH^–^ → H_2_O

**Figure 5:**
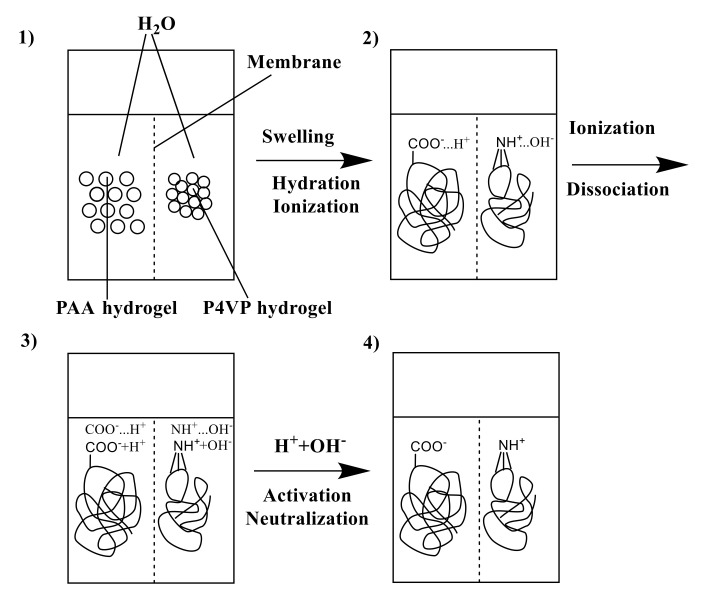
Stages of mutual activation of PAA and P4VP hydrogels.

As can be seen from these reactions, the dissociation of carboxyl groups with the consequent binding of the cleaved protons leads to a decrease in the amount of H^+^ ions in the solution. In turn, there is additional dissociation of other (undissociated) carboxyl groups (owing to Le-Chatelier principle). Such interactions lead to the formation of uncompensated charged groups on the internode links of each hydrogel, which undergo repulsion, leading to the unfolding of the polymer globe. Such transition into the ionized state of each initial macromolecule in the intergel system is called mutual activation. Stages of mutual activation of PAA and P4VP hydrogels are presented on Figure 5.

The mutual activation results in significant changes in the initial electrochemical, conformational, and sorption properties.

### 3.1. Sorption of Nd^3+^ and Sc^3+^ Ions Based on Remote Interaction

Interaction of individual initial hydrogels of PAA and P4VP and the intergel system on their basis (hPAA–hP4VP) with neodymium and scandium nitrates provides sorption of the REMs.

**Figure 6:**
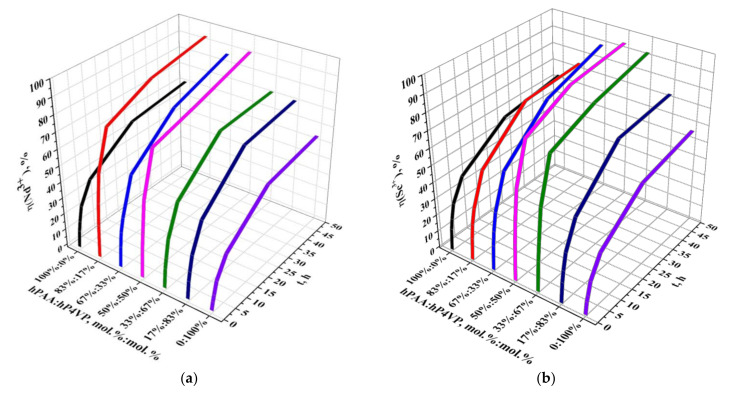
Sorption degrees of Nd^3+^ (**a**) and Sc^3+^ (**b**) by the intergel system hPAA–hP4VP.

Figure 6 presents the dependence of the sorption degrees of neodymium (a) and scandium (b) ions from molar ratios of PAA and P4VP hydrogels over time. The sorption of neodymium ions by individual polymers hPAA and hP4VP does not have a strict intense character. As seen from the figure, the sorption degree increases slightly (a sharp increase observed only during the first 6 h of interaction) with the time of the interaction of the macromolecules with the REM’s salt solution. During the time intervals of 0.5, 1, 2, and 6 h, the increase in the sorption degree is 9.81%, 15.46%, 25.35%, and 37.18% for hPAA and 4.86%, 8.49%, 15.87%, and 27.58% for hP4VP, respectively. After that, it can be said that the studied polymer hydrogels, which interact with the salt solution, are close to the equilibrium state: at 24 h of interaction, the sorption degree is 56.27% for hPAA and 48.69% for hP4VP; at 48 h, it is 61.22% and 54.15%, respectively. Higher values of the sorption degree (comparatively with individual macromolecules) in the presence of the intergel system hPAA–hP4VP point to the high ionization of the initial rare-cross-linked polymer hydrogels PAA and P4VP in the intergel pairs. Strong sorption of neodymium ions by the intergel system hPAA–hP4VP occurs during 6 h of remote interaction at the following molar ratios: 83%hPAA:17%hP4VP and 50%hPAA:50%hP4VP; these ratios are areas of maximum sorption of neodymium ions, wherein the highest values of the sorption degree are observed at the 83%hPAA:17%hP4VP ratio (the sorption degree is 93.44%). At the end of the sorption time (48 h) also, high values of the sorption degree are observed at the ratio 67%hPAA:33%hP4VP (86.57%). The maximum amount of scandium ions is sorbed by the intergel system hPAA–hP4VP at ratios 50%hPAA:50%hP4VP and 33%hPAA:67%hP4VP, wherein an overwhelming majority of scandium (more than 70%) is sorbed after 6 h of remote interaction of PAA and P4VP hydrogels in these intergel pairs; it is more than half of all the sorbed scandium; at 6 h, the sorption degree is 76.57% and 73.64%, respectively. During the same time of interaction, individual hydrogels PAA and P4VP sorb about 40% of the scandium (the sorption degree is 39.30% for hPAA and 29.70% for hP4VP). The highest values of the sorption parameter at 48 h are observed at ratios 67%hPAA:33%hP4VP (89.50%), 50%hPAA:50%hP4VP (94.24%), and 33%hPAA:67%hP4VP (92.73%).

The values of the sorption degrees of neodymium and scandium ions by the intergel system hPAA–hP4VP are presented in Table 1 and Table 2, respectively.

**Figure 7:**
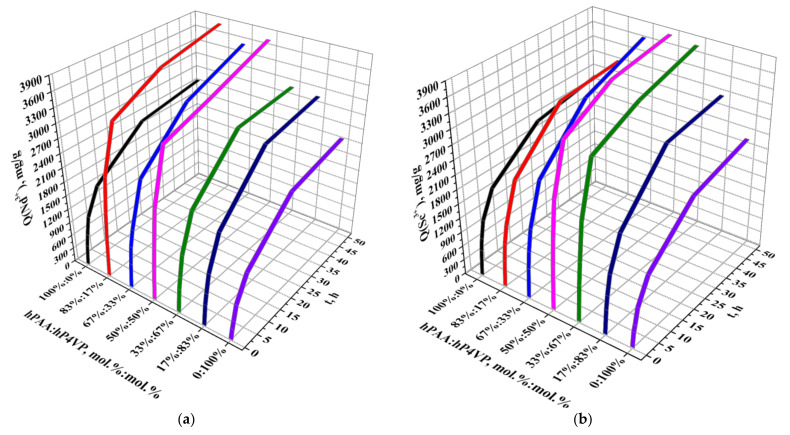
Dynamic exchange capacity (in relation to Nd^3+^ (**a**) and Sc^3+^ (**b**) ions) of the intergel system hPAA–hP4VP.

The dynamic exchange capacity (in relation to Nd^3+^ (a) and Sc^3+^ (b) ions) of the intergel system hPAA–hP4VP is shown on Figure 7. The sorption of both metals is accompanied by a significant increase in the dynamic exchange capacity of the intergel system. Strong sorption of neodymium ions is observed at the molar ratios 83%hPAA:17%hP4VP and 50%hPAA:50%hP4VP during 6 h of remote interaction, while the highest sorption values for the scandium sorption process are observed at ratios 50%hPAA:50%hP4VP and 33%hPAA:67%hP4VP at the same time interval. During this interaction time, the dynamic exchange capacity (in relation to neodymium ions) increases in the following order: 0.5 h, 821.04 mg/g; 1 h, 1380.75 mg/g; 2 h, 2108.79 mg/g; and 6 h, 3085.13 mg/g for the ratio 83%hPAA:17%hP4VP; for the ratio 50%hPAA:50%hP4VP, the parameters are 665.33, 1098.79, 1902.58, and 3009.38 mg/g, respectively. The exchange capacity (in relation to scandium ions) increases as follows: 0.5 h, 896.67 mg/g; 1 h, 1456.67 mg/g; 2 h, 2205.42 mg/g; and 6 h, 3190.42 mg/g for the molar ratio 50%hPAA:50%hP4VP. The ratio 33%hPAA:67%hP4VP has lower values of exchange capacity: 497.08, 770.42, 1166.25, and 1830.83 mg/g, respectively. The individual polymers PAA and P4VP have the following values of capacity at the same time of interaction (0.5–6 h): in relation to Nd ions, for hPAA, 408.63, 644.29, 1014.63, and 1549.08 mg/g, and for hP4VP, 202.42, 353.92, 661.13, and 1149.29 mg/g; in relation to Sc ions, for hPAA, 513.75, 753.75, 1107.08, and 1637.50 mg/g, and for hP4VP, 303.33, 429.58, 741.25, and 1237.50 mg/g. Such strong differences in the values of the sorption parameter between the intergel system and individual hydrogels is due to the high ionization of the polymers in the intergel pairs. Further interaction of the intergel system with the corresponding neodymium and scandium salt solutions leads to the consequent sorption of these metals and increase in the exchange capacity. The highest values of the exchange capacity (in relation to Nd ions) are observed at 48 h at molar ratios 83%hPAA:17%hP4VP (3893.13 mg/g) and 50%hPAA:50%hP4VP (3817.38 mg/g). The maximum values of the dynamic exchange capacity (in relation to Sc ions) at 48 h of remote interaction at molar ratios 50%hPAA:50%hP4VP and 33%hPAA:67%hP4VP are 3926.67 and 3863.75 mg/g, respectively. The sorption parameters are 2550.67 mg/g for hPAA and 2256.08 mg/g for hP4VP at Nd sorption and 2639.17 mg/g for hPAA and 2352.92 mg/g for hP4VP at Sc sorption.

Values of the dynamic exchange capacity of neodymium and scandium ions of the intergel system hPAA–hP4VP are presented in Table 3 and Table 4, respectively.

As seen from the obtained data, a significant increase in the initial sorption properties (sorption degree and dynamic exchange capacity) in the intergel system occurs due to the formation of optimal conformation for sorption of neodymium and scandium ions at certain molar ratios. Mutual activation of the rare-cross-linked polymer hydrogels during their remote interaction enables their transition into a highly ionized state.

### 3.2. Sorption of Nd^3+^ and Sc^3+^ Ions Based on Molecular Imprinting

The sorption properties (sorption degree and dynamic exchange capacity) of synthetized structures MIP(Nd) and MIP(Sc) and the control sample cMIPs are presented in Table 5 and Table 6. These data show that the sorption ability of these synthetized MIPs is sufficiently high. Strong sorption of Nd and Sc occurs due to the formation of complementary to these REM cavities in the structure of molecularly imprinted polymers during the synthesis procedure. As seen from Table 5, the sorption degree increases with time for both neodymium and scandium sorption. During 6 h of interaction, the sorption degree increases in the following order: for Nd sorption 0.5 h, 17.79%; 1 h, 29.80%; 2 h, 40.61%; and 6 h, 61.32%; for Sc sorption 0.5 h, 20.51%; 1 h, 32.94%; 2 h, 44.15%; and 6 h, 62.12%. The overwhelming majority of neodymium and scandium is sorbed during 24 h of interaction: the sorption degree of MIP(Nd) is 83.64%; the sorption degree of MIP(Sc) is 85.66%. The remaining neodymium is sorbed during the last 24 h (up to 48 h of interaction), wherein the sorption that occurs is not intense, evidenced by the fact that the sorption degree increases by 4–5%. It can be said that the system MIP–REM salt solution reaches an equilibrium state. As seen from Table 6, the dynamic exchange capacity of MIP(Nd) increases from 741.25 to 2555.00 mg/g (0.5 h, 741.25 mg/g; 1 h, 1241.67 mg/g; 2 h, 1692.08 mg/g; and 6 h, 2555.00 mg/g) during 6 h for neodymium sorption. At the same time, the increase in the capacity of MIP(Sc) during scandium sorption is 0.5 h, 854.58 mg/g; 1 h, 1372.50 mg/g; 2 h, 1839.58 mg/g; and 6 h, 2588.33 mg/g. The almost final values of the parameter for MIP(Nd), 3485.00 mg/g, and for MIP(Sc), 3569.17 mg/g, are observed at 24 h of interaction with the corresponding REM’s salt solution. Any further increase in the dynamic exchange capacity is insignificant in the case of Nd sorption by MIP(Nd); the parameter increases up to 3699.58 mg/g; in the case of Sc sorption, the capacity increases up to 3783.75 mg/g. Non-imprinted sample cMIPs do not participate in the sorption of either Nd or Sc due to the absence of complementary cavities to Nd or Sc ions.

**Figure 8:**
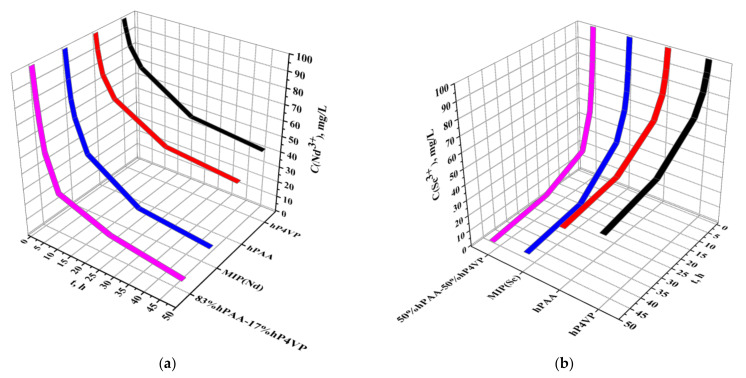
Neodymium (**a**) and scandium (**b**) concentration decrease during sorption by macromolecular sorbents.

The decrease in neodymium (a) and scandium (b) concentrations during their sorption by individual PAA and P4VP hydrogels and the intergel system and MIPs is shown in Figure 8. The figure provides comparative characteristics on the sorption efficiency of Nd or Sc ions (based on the REM concentration decrease) of the polymer structures: PAA and P4VP hydrogels, MIP(Nd)- and MIP(Sc)-imprinted polymers, and the intergel systems 83%hPAA:17%hP4VP and 50%hPAA:50%hP4VP. The character of the Nd ion concentration decrease is different for these macromolecules: the sorption of Nd ions is accompanied by the following decrease: hP4VP, 100 mg/L-95.14 mg/L-91.51 mg/L-84.13 mg/L-72.42 mg/L-51.31 mg/L-45.85 mg/L; hPAA, 100 mg/L-90.19 mg/L-84.54 mg/L-75.65 mg/L-62.83 mg/L-43.73 mg/L-38.78 mg/L; MIP(Nd), 100 mg/L-82.21 mg/L-70.20 mg/L-59.39 mg/L-38.68mg/L-16.36mg/L-11.21mg/L; 83%hPAA-17%hP4VP, 100 mg/L-80.30 mg/L-66.86 mg/L-49.39 mg/L-25.96 mg/L-13.33 mg/L-5.57 mg/L, respectively, for interaction time 0, 0.5, 1, 2, 6, 24, and 48 h. The decrease in the Sc ion concentration during sorption by these macromolecules occurs as follows: hP4VP, 100 mg/L-92.72 mg/L-89.69 mg/L-82.21 mg/L-70.30 mg/L-50.40 mg/L-43.53 mg/L; hPAA, 100 mg/L-87.67 mg/L-81.91 mg/L-73.43 mg/L-60.70 mg/L-42.02 mg/L-36.66 mg/L; MIP(Sc), 100 mg/L-79.49 mg/L-67.06 mg/L-55.85 mg/L-37.88 mg/L-14.34 mg/L-9.19 mg/L; 50%hPAA-50%hP4VP, 100 mg/L-78.48 mg/L-65.04 mg/L-47.07 mg/L-23.43 mg/L-10.40 mg/L-5.76 mg/L, respectively, for interaction time 0, 0.5, 1, 2, 6, 24, and 48 h. From these results, it can be seen that concentration decrease occurs more intensely when sorption is carried out by the intergel systems 83%hPAA:17%hP4VP and 50%hPAA:50%hP4VP and MIP(Nd)- and MIP(Sc)-imprinted polymers. Such difference in sorption intensities between these macromolecular structures and individual rare-cross-linked polymer hydrogels PAA and P4VP is due to high ionization, with the consequent formation of optimal conformation for the sorption of neodymium and scandium in these intergel systems and due to the formation of complementary cavities in the structure of the molecularly imprinted polymers during the synthesis procedure.

**Figure 9:**
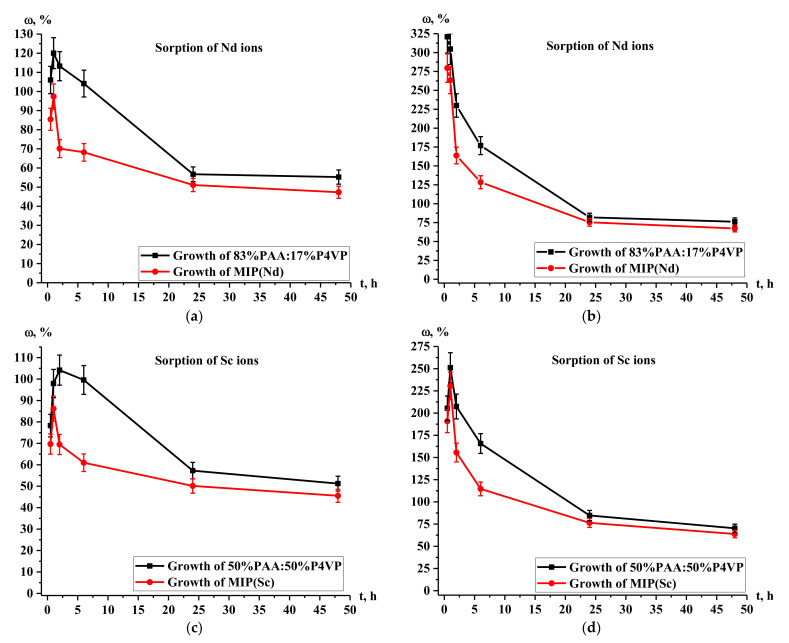
Average growth in sorption properties of intergel systems 83%hPAA:17%hP4VP and 50%hPAA:50%hP4VP and MIP(Nd) and MIP(Sc) compared to that of PAA (**a**,**c**) and P4VP (**b**,**d**) during Nd^3+^ and Sc^3+^ ion sorption.

Figure 9 presents the average growth (mean value of ω(η) and ω(Q) for the sorption of Nd and Sc ions, respectively) in the sorption properties of intergel systems 83%hPAA:17%hP4VP and 50%hPAA:50%hP4VP and MIP(Nd) and MIP(Sc) compared to those of PAA (Figure 9a,c) and P4VP (Figure 9b,d) during Nd^3+^ and Sc^3+^ ion sorption. The most significant growth in the sorption properties (in comparison with PAA hydrogel at Nd sorption) in Figure 9a occurs during 6 h for the intergel system 83%hPAA:17%hP4VP; the growth occurs as follows: 105.98% (0.5 h)-120.03% (1 h)-113.23% (2 h)-104.12% (6 h); in the same time interval, the growth in the sorption properties for MIP(Nd) occurs in the following order: 85.47% (0.5 h)-97.36% (1 h)-70.11 (2 h)-68.19 (6 h). The subsequent growth in the sorption properties is not intense: for the intergel system, it is 56.73% at 24 h and 55.26% at 48 h of remote interaction, while the growth for MIP(Nd) is 51.08% at 24 h and 47.30% at 48 h. Higher growth in sorption properties for both macromolecular structures are observed in comparison with the P4VP hydrogel (Figure 9b). The growth in the sorption properties for the intergel system 83%hPAA:17%hP4VP occurs as follows: 320.90% (0.5 h)-304.64% (1 h)-229.92% (2 h)-176.86% (6 h); at the same time, the growth for the MIP(Nd) is as follows: 279.51% (0.5 h)-263.38% (1 h)-163.74% (2 h)-128.43% (6 h); after this time, there is a growth decrease for both polymer structures: 81.89% at 24 h and 76.19% at 48 h for 83%hPAA:17%hP4VP and 75.36% (24 h) and 67.18% (48 h) for MIP(Nd). Obtained data on sorption properties’ growth in relation to scandium ions compared with that of PAA hydrogel (Figure 9c) indicate that maximum growth values are observed at 2 h of remote interaction in the presence of the intergel system 50%hPAA:50%hP4VP, wherein the growth character changes in the following order: 78.26% (0.5 h)-97.92% (1 h)-104.17% (2 h)-99.58% (6 h)-57.27% (24 h)-51.22% (48 h); the growth values for MIP(Sc) are 69.66% (0.5 h)-86.20 (1 h)-69.47% (2 h)-60.97% (6 h)-50.13% (24 h)-45.54% (48 h). The mean growth in the sorption properties compared with that of P4VP hydrogel (Figure 9d) for the intergel system 50%hPAA:50%hP4VP is 205.38% (0.5 h)-251.05% (1 h)-207.41% (2 h)-165.70% (6 h)-84.68% (24 h)-70.23 (48 h); for the imprinted polymer MIP(Sc), the growth is 190.82% (0.5 h)-230.48% (1 h)-155.58% (2 h)-114.62% (6 h)-76.34% (24 h)-63.85% (48 h). As seen from Figure 9, the growth in the sorption properties significantly increases in most cases during the first 2 h of interaction. After that, it slightly decreases up to 6 h, with a sharp decrease at 24 h. After this time, the decrease in the growth is insignificant (over 10%).

### 3.3. Laboratory Tests on Selective Sorption of Nd^3+^ and Sc^3+^ Ions

To carry out laboratory tests on the selective sorption of neodymium and scandium ions, a prototype of a laboratory unit was created. The creation of a unit prototype for the selective extraction of neodymium and scandium ions presupposes the initial development of technical requirements for the structure of the unit itself. The following basic requirements were developed:

(1) The design and elements of the unit must be resistant to aggressive environments, since testing involves interaction with strongly acidic solutions (pH = 3.5–4.5) containing ions of rare earth and rare metals.
(2) The material from which the unit is made should not enter into chemical reactions with product solutions.
(3) The unit should provide the ability to quickly change the filling polymer structures with sorbed ions of neodymium and scandium to unused ones (ready for sorption). The laboratory unit should be relatively easy to reconstruct for the use of various highly selective polymer structures: highly selective intergel systems, interpenetrating polymer networks, and molecularly imprinted polymers.

**Figure 10:**
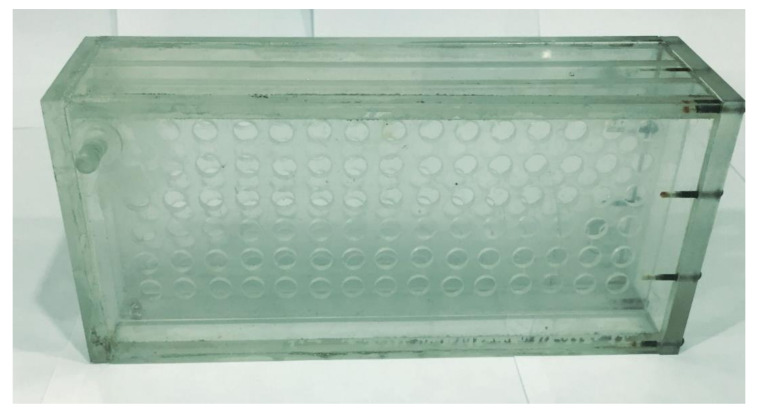
Unit for selective sorption of neodymium and scandium ions.

**Figure 11:**
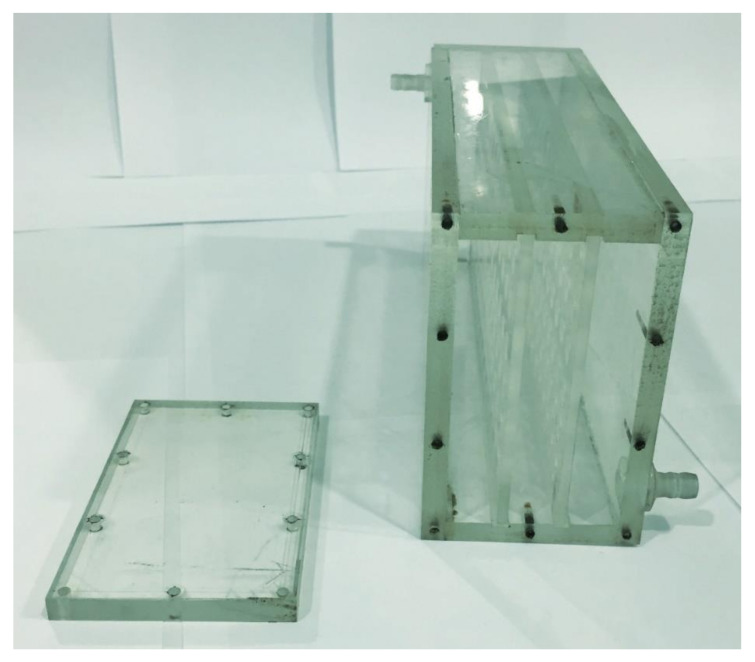
Location of cartridges inside the unit.

**Figure 12:**
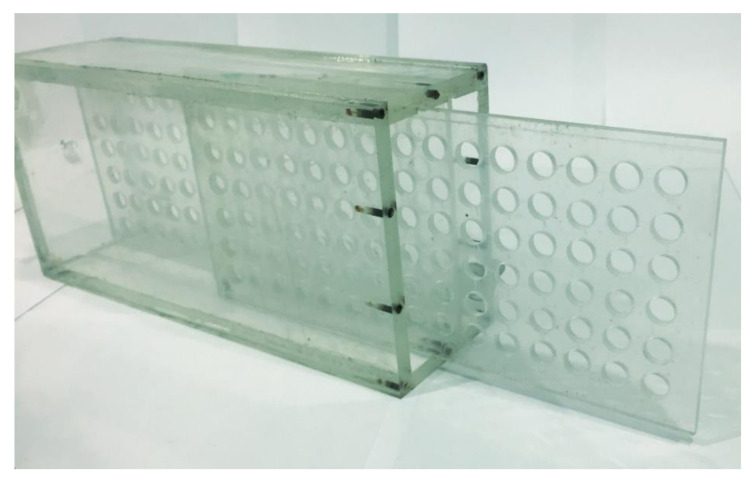
Way to quickly remove the cartridges.

Work on the design of a unit prototype installation for the selective sorption of Nd and Sc ions from industrial solutions of hydrometallurgy was carried out. Figure 10 is a photograph of an assembled unit. The laboratory unit is a structure made of plexiglass, glued with dichloroethane, containing two cartridges. The placement of the cartridges inside the unit is shown in Figure 11. Operational removal of the cartridges is shown in Figure 12 (the cartridges move along special skids back and forth). Each cartridge is covered with a special polymer membrane (the material is a polypropylene analogue of the one used for the laboratory studies). A pair of cartridges enables remote interaction of polymer structures (polyacids are placed in one cartridge and polybases in the other) in the case of using intergel systems for the sorption of Nd and Sc ions. The dimensions of the unit (outer contour) are 300 × 200 × 500 mm^3^. The size of the cartridges is 280 × 18 × 480 mm^3^. The cartridges involve the loading of functional polyacids and polybases in intergel pairs (certain molar ratios) of hPAA–hP4VP that have the maximum sorption properties relatively to neodymium and scandium ions. In addition to the above intergel systems, in the developed laboratory unit, it is possible to use molecularly imprinted polymers.

The initial results of laboratory studies showed that the maximum sorption of neodymium and scandium occurs at molar ratios 83%hPAA:17%hP4VP and 50%hPAA:50%hP4VP, respectively. High values of the sorption properties are observed in the sorption of these metals by the molecularly imprinted structures MIP(Nd) and MIP(Sc). Two pieces of the developed laboratory unit were used (one for Nd ion sorption and one for Sc ion sorption) one after another for the selective sorption and separation of neodymium and scandium ions from each other from the model solution (the solution containing neodymium nitrate hydrate and scandium nitrate hydrate, each salt at a concentration of 100 mg/L).

#### 3.3.1. Selective Sorption and Separation Based on the Intergel Systems

Table 7 and Table 8 contain values of the sorption properties (sorption degree and dynamic exchange capacity) in relation to Nd and Sc ions during the selective sorption of these ions. As can be seen from these results, the selective sorption of Nd ions by the intergel system 83%hPAA:17%hP4VP (first laboratory unit) is hampered by the accompanying sorption of scandium (Table 7). The values of the sorption degree in relation to Nd ions are as follows: 15.84% (0.5 h)-26.37% (1 h)-39.61% (2 h)-51.22% (6 h)-73.67% (24 h)-81.43% (48 h); the sorption degree of the accompanying Sc ions is 1.17% (0.5 h)-1.76% (1 h)-2.33 (2 h)-4.09% (6 h)-7.06% (24 h)-10.58% (48 h). The selective sorption of Nd ions leads to the following changes in the dynamic exchange capacity: 660.00 mg/g (0.5 h)-1098.75 mg/g (1 h)-1650.42 mg/g (2 h)-2134.17 mg/g (6 h)-3069.58 mg/g (24 h)-3392.92 mg/g (48 h); this parameter in relation to Sc ions is 48.75 mg/g (0.5 h)-73.33 mg/g (1 h)-97.08 mg/g (2 h)-170.42 mg/g (6 h)-294.17 mg/g (24 h)-440.83 mg/g (48 h). If the sorbed amount of scandium is considered to be a share of the sorbed neodymium, the mean values of the sorption properties in relation to scandium ions are 7.39% (0.5 h)-6.67% (1 h)-5.88% (2 h)-7.99% (6 h)-9.58% (24 h)-12.99% (48 h), wherein the average share of the interfering scandium during the entire time of the selective sorption of neodymium is 8.42%.

Similarly, the selective sorption of Sc ions by the intergel system 50%hPAA:50%hP4VP (second laboratory unit) is hampered by the remaining part of the sorbed neodymium (Table 8). The values of the sorption degree of scandium ions are 19.87% (0.5 h)-32.45% (1 h)-50.58% (2 h)-60.20% (6 h)-71.23% (24 h)-83.57% (48 h); the parameters in relation to neodymium ions are 2.15% (0.5 h)-2.65% (1 h)-4.33 (2 h)-7.08% (6 h)-10.01% (24 h)-13.22% (48 h). The average values of sorption properties (if the sorbed amount of scandium is considered as a share of the sorbed neodymium) in relation to neodymium ions that interfere with the selective sorption of scandium ions are as follows: 10.82% (0.5 h)-8.17% (1 h)-8.56% (2 h)-11.76% (6 h)-14.05% (24 h)-15.82% (48 h), wherein the average share of interfering neodymium during the entire time of the selective sorption of neodymium is 11.53%.

#### 3.3.2. Selective Sorption and Separation Based on Molecularly Imprinted Polymers

The sorption properties (sorption degree and dynamic exchange capacity) of the macromolecular structures MIP(Nd) and MIP(Sc) during the selective sorption of Nd and Sc ions are presented in Table 9 and Table 10. The selective sorption of neodymium ions occurs intensively during 24 h. The remaining 24 h provide an insignificant increase in the sorption properties; the sorption degree of neodymium ions increases as follows: 18.10% (0.5 h)-29.95% (1 h)-41.13% (2 h)-61.60% (6 h)-83.45% (24 h)-88.78% (48 h). The increase in the exchange capacity is 754.17 mg/g (0.5 h)-1247.92% mg/g (1 h)-1713.75 mg/g (2 h)-2566.67 mg/g (6 h)-3477.08 mg/g (24 h)-3699.17 mg/g (48 h). The selective sorption of scandium from the model solution (after neodymium sorption) points to the increase in the sorption properties; the sorption degree increases with time as follows: 21.30% (0.5 h)-33.56% (1 h)-44.69% (2 h)-62.43% (6 h)-85.78% (24 h)-90.81% (48 h). The dynamic exchange capacity increases as follows: 887.50 mg/g (0.5 h)-1398.33 mg/g (1 h)-1862.08 mg/g (2 h)-2601.25 mg/g (6 h)-3574.17 mg/g (24 h)-3783.75 mg/g (48 h).

The obtained results in the laboratory tests on selective sorption and separation showed the advantages and disadvantages of the proposed methods. The main advantage of the sorption method based on the intergel system hPAA–hP4VP is the possibility to “change” the selectivity of the system by changing molar ratios in the intergel pairs (as mentioned earlier, the maximum sorption of neodymium occurs at the ratio 83%hPAA:17%hP4VP and the maximum sorption of scandium occurs at the ratio 50%hPAA:50%hP4VP); in other words, one intergel system can be successfully applied for the selective sorption and separation of Nd and Sc ions. Nevertheless, the drawback is the accompanying sorption of Nd and Sc ions (average share of the sorbed accompanying REMs is over 10%), which, in turn, decreases the sorption properties during selective sorption and decreases the efficiency of the separation process. The advantage of the sorption method based on the imprinted polymers MIP(Nd) and MIP(Sc) is the higher values of the sorption properties (compared to that of the intergel system) and the absence of the simultaneous sorption of the accompanying metal; a drawback is that the developed MIPs are focused on the selective extraction of only one REM (each metal requires a specific MIP).

The universality of the intergel system (despite the accompanying slight sorption of the other metals) and the high selectivity of the molecularly imprinted polymers (despite the high cost and complex procedure of synthesis) are the advantages of the molecular imprinting technique that make them preferrable in the development of new-generation sorption technologies. The principle of application of molecularly imprinted polymers lies in the cost of the sorbed rare earth metal; in the case of the extraction of the most expensive rare earth metals, it would be cost effective.

## 4. Conclusions

The two developed methods for the selective sorption of Nd and Sc ions and their separation from each other showed sufficiently good results to be successfully applied in the upgrade of the existing sorption technologies. From the point of view of economical efficiency, it is more appropriate to use intergel systems for selective sorption and further separation of Nd and Sc ions despite the lower values of the sorption properties in comparison with molecularly imprinted polymers. However, there is no doubt about the absolute maximum efficiency of sorption and separation of Nd and Sc ions from each other by the application of molecularly imprinted polymers despite their complicated synthesis procedure and the high initial cost of their production.

## Figures and Tables

**Table 1:** Sorption degree of Nd^3+^ ions by the intergel system hPAA–hP4VP.

t, h	η(Nd^3+^), %						
	hPAA:hP4VP, mol.%:mol.%						
	100%:0%	83%:17%	67%:33%	50%:50%	33%:67%	17%:83%	0%:100%
0	0	0	0	0	0	0	0
0.5	9.81	19.71	12.13	15.97	11.73	10.51	4.86
1	15.46	33.14	20.92	26.37	17.58	15.87	8.49
2	24.35	50.61	29.00	45.66	28.19	25.06	15.87
6	37.18	74.04	51.12	72.23	46.17	41.52	27.58
24	56.27	86.67	73.94	80.10	69.80	66.47	48.69
48	61.22	93.44	86.57	91.62	72.02	71.11	54.15

**Table 2:** Sorption degree of Sc^3+^ ions by the intergel system hPAA–hP4VP.

T, h	η(Sc^3+^), %						
	hPAA:hP4VP, mol.%:mol.%						
	100%:0%	83%:17%	67%:33%	50%:50%	33%:67%	17%:83%	0%:100%
0	0	0	0	0	0	0	0
0.5	12.33	12.63	14.35	21.52	17.89	11.93	7.28
1	18.09	20.92	22.63	34.96	29.10	18.49	10.31
2	26.57	29.40	33.14	52.93	47.78	27.99	17.79
6	39.30	48.29	53.14	76.57	73.64	43.94	29.70
24	57.98	72.02	77.48	89.60	84.34	69.50	49.60
48	63.34	74.65	89.50	94.24	92.73	73.23	56.47

**Table 3:** Dynamic exchange capacity (in relation to Nd^3+^ ions) of the intergel system hPAA–hP4VP.

t, h	Q(Nd^3+^), mg/g						
	hPAA:hP4VP, mol.%:mol.%						
	100%:0%	83%:17%	67%:33%	50%:50%	33%:67%	17%:83%	0%:100%
0	0	0	0	0	0	0	0
0.5	408.63	821.04	505.42	665.33	488.58	438.08	202.42
1	644.29	1380.75	871.54	1098.79	732.67	661.13	353.92
2	1014.63	2108.79	1208.21	1902.58	1174.54	1044.08	661.13
6	1549.08	3085.13	2129.83	3009.38	1923.63	1730.04	1149.29
24	2344.46	3611.17	3080.92	3337.63	2908.38	2769.50	2028.83
48	2550.67	3893.13	3606.96	3817.38	3000.96	2963.08	2256.08

**Table 4:** Dynamic exchange capacity (in relation to Sc^3+^ ions) of the intergel system hPAA–hP4VP.

t, h	Q(Sc^3+^), mg/g						
	hPAA:hP4VP, mol.%:mol.%						
	100%:0%	83%:17%	67%:33%	50%:50%	33%:67%	17%:83%	0%:100%
0	0	0	0	0	0	0	0
0.5	513.75	526.25	597.92	896.67	745.42	497.08	303.33
1	753.75	871.67	942.92	1456.67	1212.50	770.42	429.58
2	1107.08	1225.00	1380.83	2205.42	1990.83	1166.25	741.25
6	1637.50	2012.08	2214.17	3190.42	3068.33	1830.83	1237.50
24	2415.83	3000.83	3228.33	3733.33	3514.17	2895.83	2066.67
48	2639.17	3110.42	3729.17	3926.67	3863.75	3051.25	2352.92

**Table 5:** Sorption degrees of Nd^3+^ and Sc^3+^ ions by the synthetized molecular-imprinted polymers.

t, h	η(Nd^3+^), %		η(Sc^3+^), %	
	MIP(Nd)	cMIP	MIP(Sc)	cMIP
0	0	0	0	0
0.5	17.79	0	20.51	0
1	29.80	0	32.94	0
2	40.61	0	44.15	0
6	61.32	0	62.12	0
24	83.64	0	85.66	0
48	88.79	0	90.81	0

**Table 6:** Dynamic exchange capacity (in relation to Nd^3+^ ions) of the synthetized molecular-imprinted polymers.

t, h	Q(Nd^3+^), mg/g		Q(Sc^3+^), mg/g	
	MIP(Nd)	cMIP	MIP(Sc)	cMIP
0	0	0	0	0
0.5	741.25	0	854.58	0
1	1241.67	0	1372.50	0
2	1692.08	0	1839.58	0
6	2555.00	0	2588.33	0
24	3485.00	0	3569.17	0
48	3699.58	0	3783.75	0

**Table 7:** Sorption properties of the intergel system hPAA–hP4VP during neodymium ion sorption.

t, h	Nd Selective Sorption			
	83%hPAA:17%hP4VP			
	η(Nd), %	Q(Nd), mg/g	η(Sc), %	Q(Sc), mg/g
0	0	0	0	0
0.5	15.84	660.00	1.17	48.75
1	26.37	1098.75	1.76	73.33
2	39.61	1650.42	2.33	97.08
6	51.22	2134.17	4.09	170.42
24	73.67	3069.58	7.06	294.17
48	81.43	3392.92	10.58	440.83

**Table 8:** Sorption properties of the intergel system hPAA–hP4VP during scandium ion sorption.

t, h	Sc Selective Sorption			
	50%hPAA:50%hP4VP			
	η(Nd), %	Q(Nd), mg/g	η(Sc), %	Q(Sc), mg/g
0	0	0	0	0
0.5	2.15	89.58	19.87	827.92
1	2.65	110.42	32.45	1352.08
2	4.33	180.42	50.58	2107.50
6	7.08	295.00	60.20	2508.33
24	10.01	417.08	71.23	2967.92
48	13.22	550.83	83.57	3482.08

**Table 9:** Sorption properties of the imprinted structures MIP(Nd) and MIP(Sc) during neodymium ion sorption.

t, h	Nd Selective Sorption			
	MIP(Nd)		MIP(Sc)	
	η(Nd), %	Q(Nd), mg/g	η(Nd), %	Q(Nd), mg/g
0	0	0	0	0
0.5	18.10	754.17	0	0
1	29.95	1247.92	0	0
2	41.13	1713.75	0	0
6	61.60	2566.67	0	0
24	83.45	3477.08	0	0
48	88.78	3699.17	0	0

**Table 10:** Sorption properties of the imprinted structures MIP(Nd) and MIP(Sc) during scandium ion sorption.

t, h	Sc Selective Sorption			
	MIP(Nd)		MIP(Sc)	
	η(Sc), %	Q(Sc), mg/g	η(Sc), %	Q(Sc), mg/g
0	0	0	0	0
0.5	0	0	21.30	887.50
1	0	0	33.56	1398.33
2	0	0	44.69	1862.08
6	0	0	62.43	2601.25
24	0	0	85.78	3574.17
48	0	0	90.81	3783.75

## Data Availability

The data presented in this study are available upon request from the corresponding author.

## References

[1] Hedrick J.B. (1999). Rare Earths: U.S. Geological Survey in Metals and Minerals in Minerals Yearbook.

[2] Cotton S. (1991). Lanthanides and Actinides.

[3] Norman A., Zou X., Barnett J. (2014). Critical Minerals: Rare Earths and the U.S. Economy.

[4] Kryukov V.A., Yatsenko V.A., Kryukov Y.V. (2020). Rare-earth industry—To realize available opportunities. Min. Ind..

[5] Kilbourn B.T. (1993). A Lanthanide Lanthology.

[6] Jones A.P., Wall F., Williams C.T. (1996). Rare Earth Minerals; Chemistry, Origin, and Ore Deposits.

[7] Lipin B.R., McKay G.A. (1989). Geochemistry and Mineralogy of the Rare Earth Elements: Reviews in Mineralogy.

[8] Bautista R.G. (1995). Separation Chemistry. Handbook on the Physics and Chemistry of Rare Earths.

[9] Alguacil F.J., Rodriguez F. (1997). Separation processes in rare earths. Rev. Metal..

[10] Larsson K., Binnemans K. (2015). Separation of rare earths by split-anion extraction. Hydrometallurgy.

[11] Innocenzi V., De Michelis I., Ferella F., Veglio F. (2016). Rare earths from secondary sources: Profitability study. Adv. Environ. Res.-Int. J..

[12] Maestro P., Huguenin D. (1995). Industrial applications of rare earths: Which way for the end of the century. J. Alloys Compd..

[13] Ryan N.E. (1995). High-Temperature Corrosion Protection. Handbook on the Physics and Chemistry of Rare Earths.

[14] Omodara L., Pitkäaho S., Turpeinen E.-M., Saavalainen P., Oravisjärvi K., Keiski R.L. (2019). Recycling and substitution of light rare earth elements, cerium, lanthanum, neodymium, and praseodymium from end-of-life applications—A review. J. Clean. Prod..

[15] Jelinek L., Wei Y.Z., Arai T., Kumagai M. (2008). Study on separation of Eu(II) from trivalent rare earths via electro-reduction and ion exchange. J. Alloys Compd..

[16] Moldoveanu G., Papangelakis V. (2021). Chelation-Assisted Ion-Exchange Leaching of Rare Earths from Clay Minerals. Metals.

[17] Rozelle P.L., Khadilkar A.B., Pulati N., Soundarrajan N., Klima M.S., Mosser M.M., Miller C.E., Pisupati S.V. (2016). A Study on Removal of Rare Earth Elements from U.S. Coal Byproducts by Ion Exchange. Metall. Mater. Trans. e-Mater. Energy Syst..

[18] Miller D.D., Siriwardane R., Mcintyre D. (2018). Anion structural effects on interaction of rare earth element ions with Dowex 50W X8 cation exchange resin. J. Rare Earths.

[19] Chandrasekara N.P.G.N., Pashley R.M. (2015). Study of a new process for the efficient regeneration of ion exchange resins. Desalination.

[20] Greenleaf J.E., SenGupta A.K. (2012). Carbon dioxide regeneration of ion exchange resins and fibers: A review. Solvent Extr. Ion Exch..

[21] Zhang J., Amini A., O’Neal J.A., Boyer T.H., Zhang Q. (2015). Development and validation of a novel modeling framework integrating ion exchange and resin regeneration for water treatment. Water Res..

[22] Melnikov Y.A., Ergozhin E.E., Chalov T.K., Nikitina A.I. (2015). The anion exchange resin based on diglycidyl benzylamine and polyethylenimine to extract perrenate ions. Int. J. Chem. Sci..

[23] Melnikov Y.A., Ergozhin E.E., Chalov T.K., Nikitina A.I. (2014). Sorption of chromium (VI) ions by anionites based on epoxidized derivatives of aniline and benzylamine. Life Sci. J..

[24] Jumadilov T.K., Kondaurov R.G., Abilov Z.A., Grazulevicius J.V., Akimov A.A. (2017). Influence of polyacrylic acid and poly-4-vinylpyridine hydrogels mutual activation in intergel system on their sorption properties in relation to lanthanum (III) ions. Polym. Bull..

[25] Jumadilov T., Abilov Z., Grazulevicius J., Zhunusbekova N., Kondaurov R., Agibayeva L., Akimov A. (2017). Mutual activation and sorption ability of rare cross-linked networks in intergel system based on polymethacrylic acid and poly-4-vinylpyridine hydrogels in relation to lanthanum ions. Chem. Chem. Technol..

[26] Nicholls I.A., Adbo K., Andersson H.S., Andersson P.O., Ankarloo J., Hedin-Dahlstrom J., Jokela P., Karlsson J.G., Olofsson L., Rosengren J. (2001). Can we rationally design molecularly imprinted polymers?. Anal. Chim. Acta.

[27] Toth B., Pap T., Horvath V., Horvai G. (2001). Which molecularly imprinted polymer is better?. Anal. Chim. Acta.

[28] Ying T.L., Gao M.J., Zhang X.L. (2001). Highly selective technique-molecular imprinting. Chin. J. Anal. Chem..

[29] Lai J.P., He X.W., Guo H.S., Liang H. (2001). A review on molecular imprinting technique. Chin. J. Anal. Chem..

[30] Haupt K., Linares A.V., Bompart M., Bernadette T.S.B. (2012). Molecularly Imprinted Polymers. Mol. Impr..

[31] Komiyama M., Mori T., Ariga K. (2018). Molecular Imprinting: Matrials Nanoarchitectonics with Molecular Information. Bull. Chem. Soc. Jpn..

[32] Chen W., Liu F., Xu Y.T., Li K.A., Tong S.Y. (2001). Molecular recognition of procainamide-imprinted polymer. Anal. Chim. Acta.

[33] Byrne M.E., Park K., Peppas N.A. (2002). Molecular imprinting within hydrogels. Adv. Drug Deliv. Rev..

[34] Yano K., Karube I. (1999). Molecularly imprinted polymers for biosensor applications. TrAC—Trends Anal. Chem..

[35] Allender C.J., Richardson C., Woodhouse B., Heard C.M., Brain K.R. (2000). Pharmaceutical applications for molecularly imprinted polymers. Int. J. Pharm..

[36] Cai W.S., Gupta R.B. (2004). Molecularly-imprinted polymers selective for tetracycline binding. Sep. Purif. Technol..

[37] Saylan Y., Yilmaz F., Ozgur E., Derazshamshir A., Yavuz H., Denizli A. (2017). Molecular Imprinting of Macromolecules for Sensor Applications. Sensors.

[38] Qiao F., Sun H., Yan H., Row K.H. (2006). Molecularly imprinted polymers for solid phase extraction. Chromatographia.

[39] Szatkowska P., Koba M., Koslinski P., Szablewski M. (2013). Molecularly Imprinted Polymers’ Applications: A Short Review. Mini-Rev. Org. Chem..

[40] Pohanka M. (2017). Sensors Based on Molecularly Imprinted Polymers. Int. J. Electrochem. Sci..

[41] Cormack P.A.G., Elorza A.Z. (2004). Molecularly imprinted polymers: Synthesis and characterisation. J. Chromatogr. B—Anal. Technol. Biomed. Life Sci..

[42] Yemis F., Alkan P., Yenigul B., Yenigul M. (2013). Molecularly Imprinted Polymers and Their Synthesis by Different Methods. Polym. Polym. Compos..

[43] Ye L. (2015). Synthetic Strategies in Molecular Imprinting. Molecularly Imprinted Polymers in Biotechnology.

[44] Balamurugan S., Spivak D.A. (2011). Molecular imprinting in monolayer surfaces. J. Mol. Recognit..

[45] Jumadilov T.K., Kondaurov R.G., Imangazy A.M., Myrzakhmetova N.O., Saparbekova I. (2019). Phenomenon of remote interaction and sorption ability of rare cross–linked hydrogels of polymethacrylic acid and poly-4-vinylpyridine in relation to erbium ions. Chem. Chem. Technol..

[46] Jumadilov T.K., Kondaurov R.G., Imangazy A.M. (2020). Features of sorption of rare-earth metals of cerium group by intergel systems based on polyacrylic acid, polymethacrylic acid and poly-4-vinylpyridine hydrogels. Bull. Karaganda Univ. Chem. Ser..

